# Associations Between Cardiac Function and Brain Health in Diverse Middle-Aged Adults: The Dallas Heart Study-2

**DOI:** 10.1016/j.jacadv.2023.100777

**Published:** 2023-12-22

**Authors:** John M. Giacona, Ricardo Chia, Weerapat Kositanurit, Jijia Wang, Colby Ayers, Ambarish Pandey, Julia Kozlitina, Mark H. Drazner, Sonia Garg, James A. de Lemos, Rong Zhang, Ihab Hajjar, Frank F. Yu, Laura Lacritz, Wanpen Vongpatanasin

**Affiliations:** a Hypertension Section, Department of Internal Medicine, University of Texas Southwestern Medical Center, Dallas, Texas, USA; b Department of Applied Clinical Research, University of Texas Southwestern Medical Center, Dallas, Texas, USA; c Department of Physiology, Faculty of Medicine, Chulalongkorn University, Bangkok, Thailand; d Cardiology Division, University of Texas Southwestern Medical Center, Dallas, Texas, USA; e Eugene McDermott Center for Human Growth and Development, University of Texas Southwestern Medical Center, Dallas, Texas, USA; f Department of Neurology and Neurotherapeutics, University of Texas Southwestern Medical Center, Dallas, Texas, USA; g Department of Radiology, University of Texas Southwestern Medical Center, Dallas, Texas, USA; h Department of Psychiatry, University of Texas Southwestern Medical Center, Dallas, Texas, USA

**Keywords:** Alzheimer disease, cardiac function, cardiac structure, cognitive function, dementia, race

## Abstract

**Background:**

Previous studies have linked cardiovascular risk factors during midlife to cognitive function in later life. However, few studies have looked at the association between cardiac function, brain structure, and cognitive function and even less have included diverse middle-aged populations.

**Objectives:**

The objective of this study was to determine associations between cardiac and brain structure and function in a multiethnic cohort of middle-aged adults.

**Methods:**

A cross-sectional study was conducted in participants of the Dallas Heart Study phase 2 (N = 1,919; 46% Black participants). Left ventricular (LV) mass, LV ejection fraction, LV concentricity, and peak systolic strain (LV E_cc_) were assessed by cardiac magnetic resonance imaging. White matter hyperintensities (WMH) volume was measured by fluid attenuated inversion recovery magnetic resonance imaging. The Montreal Cognitive Assessment was used to measure cognitive functioning. Associations between cardiac and brain measures were determined using multivariable linear regression after adjusting for cardiovascular risk factors, education level, and physical activity.

**Results:**

LV ejection fraction was associated with total Montreal Cognitive Assessment score (β = 0.06 [95% CI: 0.003-0.12], *P* = 0.042) and LV E_cc_ was associated with WMH volume (β = 0.08 [95% CI: 0.01-0.14], *P* = 0.025) in the overall cohort without significant interaction by race/ethnicity. Higher LV mass and concentricity were associated with larger WMH volume in the overall cohort (β = 0.13 [95% CI: 0.03-0.23], *P* = 0.008 and 0.10 [95% CI: 0.03-0.17], *P* = 0.005). These associations were more predominant in Black than White participants (β = 0.17 [95% CI: 0.04-0.30] vs β = −0.009 [95% CI: −0.16 to 0.14], *P* = 0.036 and β = 0.22 [95% CI: 0.13-0.32] vs β = −0.11 [95% CI: −0.21 to −0.01], *P* < 0.0001, for LV mass and concentricity, respectively).

**Conclusions:**

Subclinical cardiac dysfunction indicated by LVEF was associated with lower cognitive function. Moreover, LV mass and concentric remodeling were associated with higher WMH burden, particularly among Black individuals.

Over the past few decades, there has been growing evidence supporting the association between cardiac dysfunction and impaired cognitive function in older adults.^1^, ^2^, ^3^ It was estimated that patients with congestive heart failure experienced 2- to 4-fold higher risk of cognitive impairment than age-matched controls.^4^^,^^5^ This association between cardiac and brain function was observed not only in patients with overt heart failure but also in community-dwelling older adults without overt cardiovascular disease.^6^^,^^7^ However, these studies to date have been conducted mainly in older populations after the sixth decade of life, who are more susceptible to develop cognitive impairment from Alzheimer disease or vascular dementia.^1^, ^2^, ^3^^,^^6^, ^7^, ^8^ In addition, recent studies have demonstrated a paradoxically lower brain amyloid burden among Black individuals based on positron emission tomography imaging when compared to White individuals despite having a higher prevalence of the apolipoprotein E4 allele (ApoE-ε4) and prevalence of dementia.^9^^,^^10^ These finding suggested a complex pathophysiologic mechanisms of dementia that is not explained by brain amyloid alone, which may contribute to racial/ethnic disparities in prevalence of dementia.

There are few studies that have investigated the association between cardiac and brain structure and function, especially in middle-aged individuals. Moreover, previous studies have not examined the potential role of race/ethnicity in modifying the association between cardiac structure and function and cognitive function in diverse middle-aged populations. Accordingly, we performed a cross-sectional study in the young to middle-aged multiethnic population enrolled in the DHS-2 (Dallas Heart Study phase 2) to determine associations between cardiac function and brain structure and function, after adjusting for traditional vascular risk factors as well as physical activity.

## Methods

**Figure 1:**
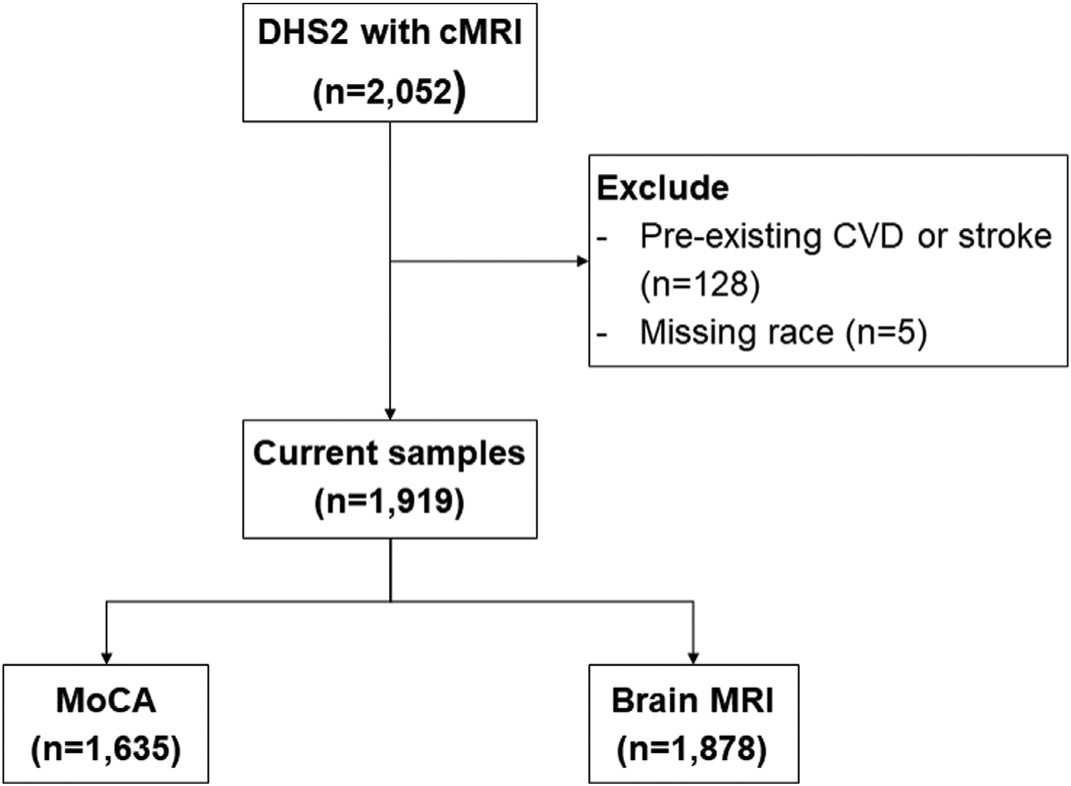
**Study FlowChart**

Studies were conducted in participants enrolled in DHS-2 between 2007 and 2009 (NCT00344903) after informed consent was obtained. The study was approved by the Institutional Review Board at the University of Texas Southwestern Medical Center. Details regarding DHS-2 selection criteria, study design, and methods have been described elsewhere.^11^ The Dallas Heart Study is a multiethnic, population-based probability sample of Dallas County. DHS-2 participants underwent cognitive testing, brain magnetic resonance imaging (MRI) and cardiac magnetic resonance imaging (cMRI), and physical activity assessment during 1 visit. Race was self-reported by study participants, and race categories were defined by investigators based on the US Office of Management and Budget’s Revisions to the Standards for the Classification of Federal Data on Race and Ethnicity. As shown in Figure 1, a total of 2,052 participants underwent cMRI; in which, 128 participants with prior history of cardiovascular disease or stroke and 5 participants without reporting race/ethnicity were excluded. Therefore, 1,919 participants were included in our current analysis. Of those, 1,635 participants underwent cognitive function testing with the Montreal Cognitive Assessment (MoCA) and 1,878 participants completed brain MRI.

### Cardiac magnetic resonance imaging

cMRI was performed on a 3-T MRI system (Achieva, Philips Medical Systems).^12^ Left ventricular (LV) images were acquired using prospective electrocardiography gating and turbo field echo sequencing. LV mass, LV end-diastolic volume, LV end-systolic volume, left atrial volume (LA), LV ejection fraction (LVEF), cardiac output, heart rate, blood pressure (BP), and stroke volume were calculated as previously described.^13^ LV concentricity was defined as LV mass/LV end-diastolic volume (EDV)^0.67^ as previously described.^14^ Peak systolic strain (LV E_cc_) was assessed using the myocardial tissue tagging method in 6 LV wall segments using harmonic phase imaging software offline (HARP, Diagnosoft Virtue 5.04, Diagnosoft) as previously described.^15^

### Montreal cognitive assessment

The total MoCA score is a 30-point screening tool for the assessment of cognitive function^16^ that includes assessment of attention, orientation, language, verbal memory, visuospatial, and executive function.^17^ The MoCA was administered by trained personnel in DHS-2 as previously described.^16^

### Brain MRI

A 3-T MRI unit (Achieva, Philips Medical Systems) was used for the DHS brain MRI protocol, with acquisition of 2-dimensional fluid-attenuated inversion recovery images and 3-dimensional magnetization prepared rapid acquisition with gradient echo images as previously detailed.^18^ Only structural brain imaging was acquired in DHS-2, regional quantification of brain volumes was performed using the freely available Functional MRI of the Brain Software Library.^18^, ^19^, ^20^ Measurements of white matter hyperintensities (WMH), and total cranial volumes were extracted as previously described. No functional MRI was available or processed for this study.

### Apolipoprotein E genotyping

Genomic DNA was extracted from circulating leukocytes. TaqMan single-nucleotide polymorphism genotyping assays from Applied Biosystems were used to genotype c.388T>C (rs429358) and c.526C>T (rs7412), that define 3 haplotypes ε2(388T-526T), ε3(388T-526C), and ɛ4(388C-526C). ApoE-ε4 allele positive carrier status is defined as ε2/ε4, ε3/ε4, or ε4/ε4 and negative as ε2/ε2, ε2/ε3, or ε3/ε3.

### Assessment of physical activity

Time spent in moderate-to-vigorous physical activity was assessed using a wrist-based accelerometer (Actical, Philips Respironics) in participants enrolled in DHS-2 between 2008 and 2009 as previously described.^11^

### Statistical analysis

Statistical analyses were performed using SAS, version 9.4. Participant characteristics are reported as mean ± SD, or median (IQR) for skewed data, and number (percentage) for categorical data. Two-sample t-test and Mann-Whitney U test were used to compare baseline continuous measurements and Chi-square test was used to compare baseline categorical measurements. To address association between cardiac function and brain function, the primary exposure is LVEF, and primary outcome is total MoCA scores. Multivariable linear regression analyses were performed to assess the associations of markers of cardiac structure and function with total MoCA scores. Similarly, to address the association between cardiac structure and brain structure, the primary exposure is LV mass and the primary outcome is WMH. Multivariable linear regression analyses were also performed to determine the associations of markers of cardiac structure and function with brain WMH volume after normalizing to total cranial volumes. All modeling assumptions were verified using diagnostic tools (such as residual vs predicted value plot, Q-Q plot, and Cook’s distance). Since the WMH data were skewed, the analysis was performed after a log transformation. All models were adjusted for race/ethnicity, and interactions between race/ethnicity and risk factors were tested by including multiplicative interaction terms in the model. Three multivariable models were assembled and included the following covariates: 1) age, sex, race/ethnicity, and body surface area; 2) all covariates from model 1 plus systolic BP, antihypertensive drug use, diabetes mellitus, current smoking, estimated glomerular filtration rate, and education levels; and 3) model 2 covariates plus time spent in moderate-to-vigorous physical activity. Education levels (model 2) were categorized into 4 levels: less than high school education, high school education, college, and advanced degree above college degree, and were included as a categorical variable in model 2. Similar multivariable models were assembled to additionally include atrial fibrillation as a covariate in model 2. Restricted cubic splines of each association between cardiac and brain variables were also constructed using model 3. Statistical significance was indicated when *P* value is <0.05.

To determine if the relationship between cardiovascular variables and cognitive function was dependent on genetic predisposition to Alzheimer disease, we further stratified participants by the ApoE-ε4 allele carrier status and self-identified race (Black participants vs non-Black participants).

## Results

### Participant characteristics

The baseline characteristics of the study participants are shown in Table 1. Of those 1,919 participants, the mean age was 49.8 ± 10.7 years, and the mean body mass index was 29.6 ± 5.6 kg/m^2^. Overall, 58.7% were women and 46.3% were Black participants. Postsecondary education levels (college and above) were reported by 64.2% of participants. Approximately 31.8% of participants were ApoE-ε4 carriers. The proportion of female participants, body mass index, systolic BP, diastolic BP, heart rate, and stroke volume was significantly higher in Black than in non-Black participants (Table 1). LVEF was significantly lower in Black than non-Black individuals, while peak systolic strain was not significantly different between the 2 groups. Higher proportion of diabetes, current smoking, and ApoE-ε4 carrier, as well as lower proportion of antihypertensive drug use and education above college were identified among Black than non-Black participants (Table 1).

**Table 1:** Baseline Characteristics of the Cohort

	Total (N = 1,919)	Black (n = 888)	Non-Black (n = 1,031)	*P* Value
Age (y)	49.8 ± 10.7	49.3 ± 10.8	50.2 ± 10.5	0.074
Female	58.7	64.6	53.6	<0.0001^a^
BMI (kg/m^2^)	29.6 ± 5.6	30.4 ± 5.9	28.8 ± 5.2	<0.0001^a^
eGFR (mL/min/1.73 m^2^)	59.4 ± 3.9	59.3 ± 4.8	59.5 ± 2.7	0.137
Systolic blood pressure (mm Hg)	130.5 ± 18.5	134.9 ± 19.7	126.7 ± 16.6	<0.0001^a^
Diastolic blood pressure (mm Hg)	80.3 ± 8.9	82.5 ± 9.3	78.4 ± 8.1	<0.0001^a^
Antihypertensive medication	55.7	44.9	65.1	<0.0001^a^
Diabetes mellitus (%)	12.7	15.1	10.6	0.003^a^
Current smoker (%)	19.8	23.9	16.2	<0.0001^a^
Education status				<0.0001^a^
Less than high school	13.1	11.0	14.8	
High school	22.7	30.8	15.7	
College level	53.8	52.3	55.1	
Above college	10.4	5.9	14.4	
ApoE-ε4 carrier	31.8	38.7	26.4	<0.0001^a^
MVPA (min/d)	40.9 ± 36	38.2 ± 35.9	43.3 ± 35.9	<0.0001^a^
Left ventricular ejection fraction (%)	69.0 ± 6.4	68.6 ± 6.9	69.2 ± 6.0	0.035^a^
Peak systolic strain (%)	−14.5 ± 2.9	−14.5 ± 3.0	−14.5 ± 2.7	0.887
Heart rate (beats/min)	66.3 ± 10.2	66.9 ± 10.3	65.8 ± 10.0	0.013^a^
Stroke volume (ml)	81.1 ± 16.3	83.1 ± 16.9	79.4 ± 15.6	<0.0001^a^

Values are mean ± SD or %.

BMI = body mass index; eGFR = estimated glomerular filtration rate; MVPA = moderate-to-vigorous physical activity.

a Indicates statistical significance.

### Association between cardiac structure/function and cognitive function

**Figure 2:**
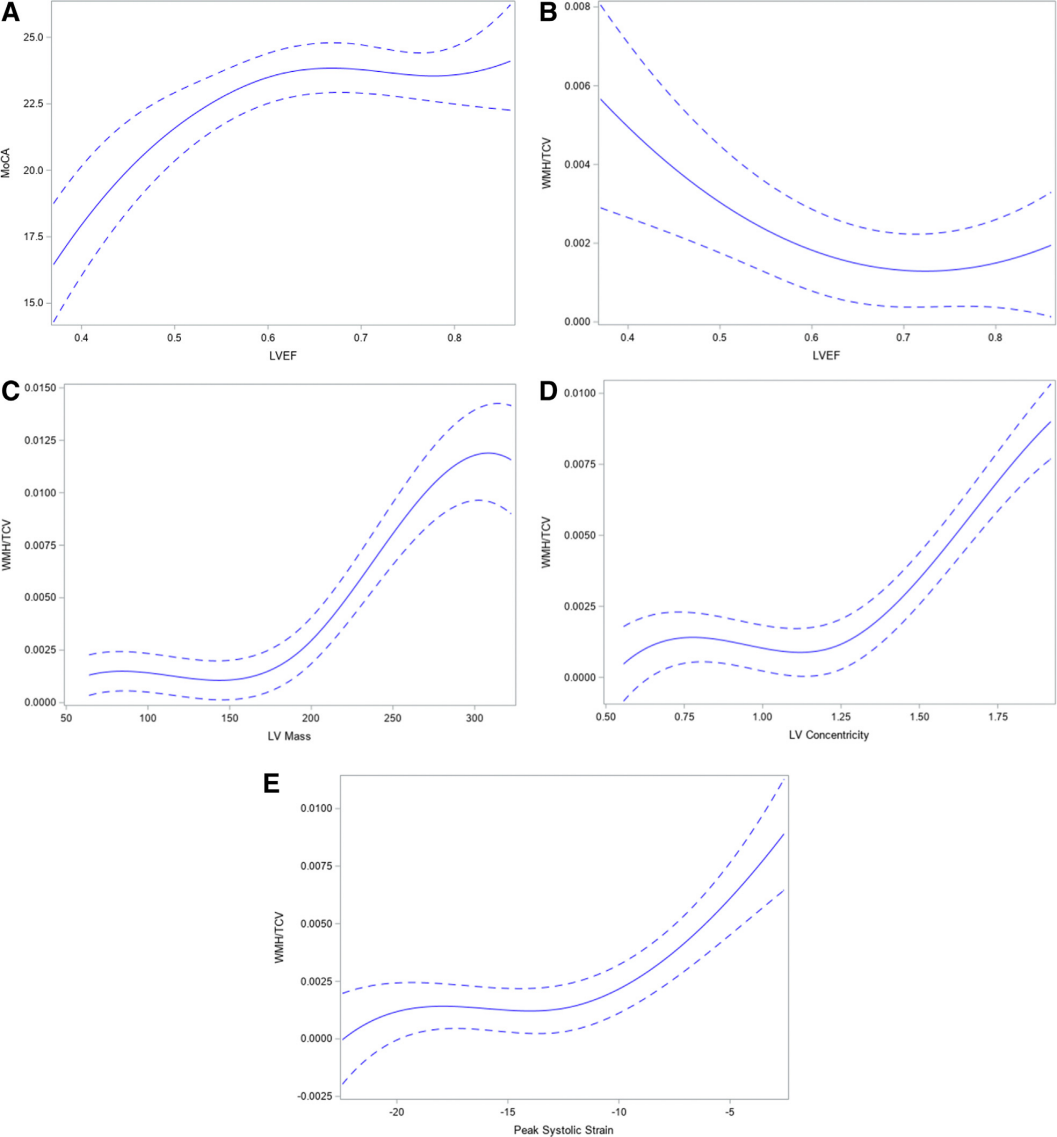
Association Between Cardiac and Brain Structure and Function Restricted cubic spline and 95% confidence band showing (A) association between left ventricular ejection fraction (LVEF) and total Montreal Cognitive Assessment (MoCA) score and association between (B) LVEF, (C) left ventricular (LV) mass, (D) LV concentricity, and (E) peak systolic strain (LV E_cc_) and white matter hyperintensities (WMH) normalized by total cranial volume (TCV).

Higher LVEF was associated with higher total MoCA score after adjustment for cardiovascular risk factors, education, and time spent in moderate-to-vigorous physical activity (β = 0.06 [95% CI: 0.003-0.12], *P* = 0.042 (Table 2, Figure 2A, and Central Illustration). There were no associations between cardiac output, LV mass, LV concentricity index, LA volume, or LV E_cc_ with total MoCA score. These findings remained the same when atrial fibrillation was added as a covariate in model 2 (Supplemental Table 1).

**Table 2:** Associations Between Cardiac Structure/Function and Total Montreal Cognitive Assessment Score (N = 1,639)

	Model 1		Model 2		Model 3	
	β (95% CI)^a^	*P* Value	β (95% CI)^a^	*P* Value	β (95% CI)^a^	*P* Value
LVEF	0.01 (−0.04 to 0.06)	0.652	0.05 (−0.01 to 0.10)	0.115	0.06 (0.003-0.12)	0.042^b^
LAV	−0.04 (−0.12 to 0.05)	0.400	0.13 (0.009-0.25)	0.037^b^	0.11 (−0.02 to 0.24)	0.105
Cardiac output	−0.009 (−0.06 to 0.04)	0.737	0.01 (−0.06 to 0.08)	0.737	0.01 (−0.06 to 0.08)	0.766
LV concentricity	−0.02 (−0.07 to 0.03)	0.499	−0.03 (−0.10 to 0.03)	0.299	−0.02 (−0.08 to 0.05)	0.572
Stroke Volume	0.01 (−0.04 to 0.07)	0.705	0.07 (0.003-0.14)	0.040^b^	0.07 (−0.01 to 0.14)	0.072
EDV	−0.003 (−0.06 to 0.05)	0.925	0.02 (−0.05 to 0.09)	0.524	0.006 (−0.07 to 0.08)	0.871
ESV	−0.01 (−0.07 to 0.04)	0.587	−0.03 (−0.10 to 0.03)	0.314	−0.05 (−0.12 to 0.02)	0.132
HR	−0.03 (−0.07 to 0.02)	0.234	−0.05 (−0.11 to 0.005)	0.074	−0.05 (−0.11 to 0.01)	0.103
LV mass	−0.008 (−0.07 to 0.06)	0.802	−0.009 (−0.09 to 0.08)	0.841	−0.006 (−0.10 to 0.08)	0.894
LVH (by BSA)	−0.007 (−0.05 to 0.04)	0.769	−0.02 (−0.07 to 0.04)	0.602	−0.03 (−0.09 to 0.03)	0.342
Peak systolic strain	−0.04 (−0.09 to 0.007)	0.096	−0.06 (−0.11 to 0.004)	0.066	−0.05 (−0.11 to 0.01)	0.101

Model 1: Age, sex, race, BSA. Model 2: Model 1 + SBP, antihypertensive, diabetes, smoking, eGFR, and education levels. Model 3: Model 2 + MVPA.

BSA = body surface area; EDV = end-diastolic volume; ESV = end-systolic volume; HR = heart rate; LAV = left atrial volume; LV = left ventricle; LVEF = left ventricular ejection fraction; LVH = left ventricular hypertrophy; MVPA = moderate-to-vigorous physical activity.

a β and 95% CI values are presented as standardized β.

b Indicates statistical significance.

**Central Illustration:**
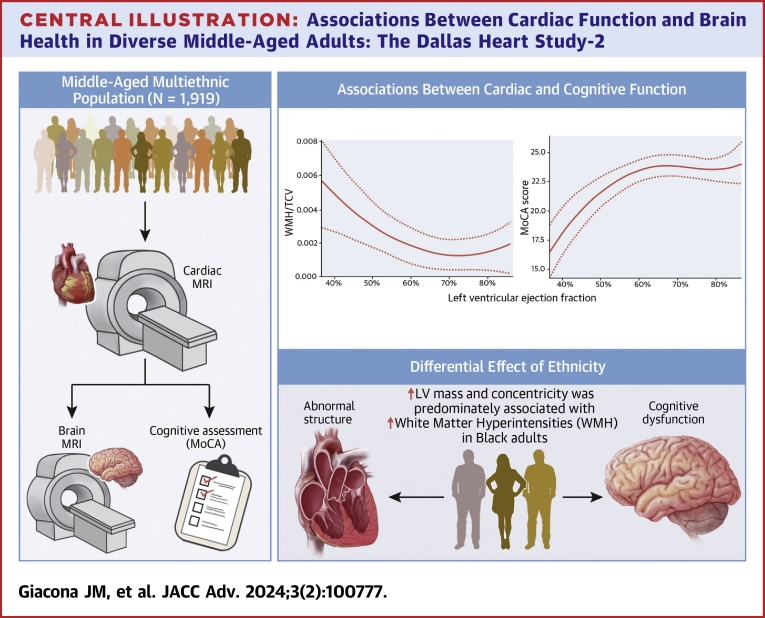
**Associations Between Cardiac Function and Brain Health in Diverse Middle-Aged Adults: The Dallas HeartStudy-2**

### Association between cardiac structure/function and brain structure

Lower LVEF was associated with higher WMH volume after adjustment for cardiovascular risk factors and physical activity (β = −0.08 [95% CI: −0.14 to −0.01], *P* = 0.024) (Table 3, Figure 2B). Higher LV mass, prevalent LV hypertrophy, LV concentricity, and higher LV E_cc_ (which generally reflected lower LV systolic function), were also associated with higher WMH volume after normalizing to total cranial volumes in the fully adjusted model (β = 0.13 [95% CI: 0.03-0.23], 0.11 [95% CI: 0.04-0.17], 0.10 [95% CI: 0.03-0.17], and 0.08 [95% CI: 0.01-0.14], respectively, all *P* < 0.05) (Table 3, Figures 2C to 2E). There was no correlation between stroke volume, cardiac output, LA volume, or heart rate with WMH volume. These relationships remain unaltered in model 3 when atrial fibrillation was added as a covariate in model 2 (Supplemental Table 2).

**Table 3:** Associations Between White Matter Hyperintensity Volume Normalized to Total Cranial Volume and Cardiac Structure/Function (N = 1,882)

	Model 1		Model 2		Model 3	
	β (95% CI)^a^	*P* Value	β (95% CI)^a^	*P* Value	β (95% CI)^a^	*P* Value
LVEF	−0.03 (−0.07 to 0.01)	0.163	−0.06 (−0.12 to −0.0009)	0.047^b^	−0.08 (−0.14 to −0.01)	0.024^b^
LAV	0.01 (−0.06 to 0.09)	0.753	−0.09 (−0.21 to 0.04)	0.195	−0.06 (−0.19 to 0.08)	0.421
Cardiac output	0.06 (0.02-0.11)	0.009^b^	0.02 (−0.06 to 0.09)	0.678	−0.004 (−0.08 to 0.07)	0.913
LV concentricity	0.10 (0.06-0.14)	<0.0001^b^	0.08 (0.02-0.15)	0.013^b^	0.10 (0.03-0.17)	0.005^b^
Stroke Volume	0.03 (−0.02 to 0.08)	0.229	−0.04 (−0.11 to 0.04)	0.343	−0.04 (−0.12 to 0.04)	0.381
EDV	0.05 (−0.001 to 0.10)	0.057	0.006 (−0.07 to 0.08)	0.876	0.02 (−0.06 to 0.10)	0.681
ESV	0.05 (−0.00007 to 0.09)	0.051	0.04 (−0.02 to 0.11)	0.200	0.06 (−0.01 to 0.13)	0.104
HR	0.04 (−0.003 to 0.08)	0.068	0.05 (−0.007 to 0.11)	0.086	0.04 (−0.03 to 0.11)	0.264
LV mass	0.17 (0.11-0.23)	<0.0001^b^	0.09 (0.004-0.18)	0.042^b^	0.13 (0.03-0.23)	0.008^b^
LVH (by BSA)	0.11 (0.07-0.15)	<0.0001^b^	0.11 (0.05-0.17)	0.0002^b^	0.11 (0.04-0.17)	0.002^b^
Peak systolic strain	0.07 (0.03-0.11)	0.0005^b^	0.06 (0.0008-0.12)	0.047^b^	0.08 (0.01-0.14)	0.025^b^

Model 1: Age, sex, race, BSA. Model 2: Model 1 + SBP, antihypertensive, diabetes mellitus, smoking, eGFR, and education levels. Model 3: Model 2 + time spent in MVPA.

BSA = body surface area; EDV = end-diastolic volume; ESV = end-systolic volume; HR = heart rate; LAV = left atrial volume; LV = left ventricle; LVEF = left ventricular ejection fraction; LVH = left ventricular hypertrophy; MVPA = moderate-to-vigorous physical activity.

a β and 95% CI values are presented as standardized β.

b Indicates statistical significance.

### Interaction with race and ethnicity and ApoE4 carrier status

Association between LVEF and MoCA was not modified by race and ethnicity (Table 4), but LV E_cc_ was inversely associated with MoCA in Black but not in non-Black individuals (β = −0.43 [95% CI: −0.75 to −0.12], *P* for interaction = 0.009, Table 4). LV E_cc_ was positively associated with WMH volume (β = 0.08 [95% CI: 0.01-0.14], *P* = 0.025) (Table 3) in the overall cohort without significant interactions by race/ethnicity (Table 5). In contrast, associations of higher LV mass and concentricity with higher WMH volume were modified by race/ethnicity, with stronger associations observed in Black individuals (β = 0.78 [95% CI: 0.46-1.11] and β = 0.31 [95% CI: 0.02-0.60], *P* for interaction <0.05 for both) (Table 5). These findings remain unaltered when atrial fibrillation was added as a covariate in model 2 (Supplemental Tables 3 and 4). Additionally, relationships between LVEF, LV mass, concentricity, and LV E_cc_ with MoCA score and WMH were not modified by ApoE-ε4 allele carrier status (Supplemental Tables 5 and 6). These findings remained the same when atrial fibrillation was added as a covariate in model 2 (Supplemental Tables 7 and 8).

**Table 4:** Association Between Cardiac Structure/Function and Montreal Cognitive Assessment Stratified by Race and Ethnicity (Adjusted for Model 3 Covariates)

	Black (n = 762)		Non-Black (n = 873)		Interaction *P* Value
	β (95% CI)^a^	*P* Value	β (95% CI)^a^	*P* Value	
LVEF	0.08 (−0.01 to 0.18)	0.083	0.04 (−0.06 to 0.13)	0.444	0.583
LAV	0.18 (0.009-0.36)	0.042^b^	−0.08 (−0.30 to 0.13)	0.457	0.336
Cardiac output	0.02 (−0.09 to 0.13)	0.767	−0.008 (−0.11 to 0.10)	0.884	0.547
LV concentricity	−0.06 (−0.16 to 0.04)	0.226	0.03 (−0.07 to 0.13)	0.569	0.592
Stroke Volume	0.08 (−0.04 to 0.19)	0.185	0.05 (−0.07 to 0.16)	0.424	0.651
EDV	−0.02 (−0.13 to 0.09)	0.734	0.04 (−0.08 to 0.15)	0.539	0.704
ESV	−0.09 (−0.19 to 0.01)	0.084	0.009 (−0.09 to 0.11)	0.866	0.410
HR	−0.05 (−0.14 to 0.04)	0.302	−0.05 (−0.15 to 0.04)	0.271	0.788
LV mass	−0.08 (−0.21 to 0.05)	0.236	0.07 (−0.07 to 0.22)	0.301	0.690
LVH (by BSA)	−0.07 (−0.17 to 0.02)	0.142	0.03 (−0.06 to 0.12)	0.513	0.423
Peak systolic strain	−0.15 (−0.24 to −0.05)	0.004^b^	0.05 (−0.04 to 0.14)	0.260	0.009^b^

Covariates: Age, sex, BSA, SBP, antihypertensive, diabetes, smoking, eGFR, and education levels, and MVPA.

BSA = body surface area; EDV = end-diastolic volume; ESV = end-systolic volume; HR = heart rate; LAV = left atrial volume; LV = left ventricle; LVEF = left ventricular ejection fraction; LVH = left ventricular hypertrophy; MVPA = moderate-to-vigorous physical activity.

a β and 95% CI values are presented as standardized β.

b indicates statistical significance.

**Table 5:** Association Between Cardiac Structure/Function and White Matter Hyperintensity Volume Normalized to Total Cranial Volume Stratified by Race and Ethnicity (Adjusted for Model 3 Covariates)

	Black		Non-Black		Interaction *P* Value
	β (95% CI)^a^	*P* Value	β (95% CI)^a^	*P* Value	
LVEF	−0.11 (−0.20 to −0.02)	0.021^b^	−0.01 (−0.11 to 0.08)	0.798	0.184
LAV	−0.11 (−0.30 to 0.08)	0.251	0.1 (−0.11 to 0.32)	0.355	0.858
Cardiac output	−0.11 (−0.22 to −0.001)	0.047^b^	0.11 (0.005-0.22)	0.040^b^	0.052
LV concentricity	0.22 (0.13-0.32)	<0.0001^b^	−0.11 (−0.21 to −0.01)	0.031^b^	<0.0001^b^
Stroke Volume	−0.17 (−0.27 to −0.06)	0.003^b^	0.13 (0.02-0.25)	0.023^b^	0.022^b^
EDV	−0.08 (−0.19 to 0.03)	0.176	0.14 (0.02-0.25)	0.024^b^	0.148
ESV	0.04 (−0.06 to 0.14)	0.488	0.08 (−0.03 to 0.18)	0.154	0.778
HR	0.04 (−0.05 to 0.14)	0.407	0.02 (−0.08 to 0.11)	0.702	0.922
LV mass	0.17 (0.04-0.30)	0.010^b^	−0.009 (−0.16 to 0.14)	0.901	0.036^b^
LVH (by BSA)	0.14 (0.04-0.23)	0.005^b^	−0.0001 (−0.09 to 0.09)	0.998	0.327
Peak systolic strain	0.11 (0.01-0.21)	0.029^b^	0.02 (−0.08 to 0.11)	0.715	0.138

Covariates: Age, sex, BSA, SBP, antihypertensive, diabetes, smoking, eGFR, and education levels, and MVPA.

BSA = body surface area; EDV = end-diastolic volume; ESV = end-systolic volume; HR = heart rate; LAV = left atrial volume; LV = left ventricle; LVEF = left ventricular ejection fraction; LVH = left ventricular hypertrophy; MVPA = moderate-to-vigorous physical activity.

a β and 95% CI values are presented as standardized β.

b indicates statistical significance.

## Discussion

The main findings from our study are 3-fold. First, in this multiethnic cohort of predominantly middle-aged adults without preexisting cardiovascular disease or stroke, lower LVEF was associated with lower cognitive function. Second, lower LVEF, higher LV mass and concentricity, and higher LV E_cc_ were associated with larger WMH volume, a marker of small vessel cerebrovascular disease and brain aging.^21^^,^^22^ Third, the magnitude of association between abnormal LV structure and WMH was stronger in Black than in non-Black adults.

Mechanisms linking cardiac structure and function with WMH and cognitive function are unknown. Although lower cerebral blood flow may be responsible for the associations between cardiac performance and brain structure and function, cerebral autoregulation normally maintains cerebral blood flow at a constant level under normal physiological conditions. Mechanisms underlying higher WMH associated with LV hypertrophy or hypertrophic cardiac remodeling are also unknown. WMH are thought to represent microvascular disease, which is commonly identified in older adults with hypertension, diabetes, stroke, or dementia.^22^ In our study, although the association between abnormal cardiac structure and WMH remained significant after adjustment for BP and other cardiovascular risk factors, it is possible that cumulative effects of persistent hypertension promote both LV hypertrophy and hypertensive related brain injury that is not otherwise captured by BP assessment at a single time point. Alternatively, hypotension may likewise contribute to WMH.^23^^,^^24^ Though, it is difficult to assess in our study because cognitive testing, brain, and cardiac MRI were done during an in-person visit in hemodynamically stable patients who were largely asymptomatic. Moreover, atrial fibrillation is a known risk factor for cognitive impairment and dementia. Some mechanisms underlying the observed associations are thought to be related to cerebral infarcts, cerebral hemorrhage, or cerebral hypoperfusion.^25^, ^26^, ^27^ However, when history of atrial fibrillation was incorporated into the models in our study, the observed relationship between cardiac structure/function with WMH and cognitive function remain the same.

Several population studies have demonstrated an association between cardiac function and brain function but almost all studies have focused on the association during late life. Analysis from the Framingham Heart Study Offspring Cohort showed a U-shape relationship between LVEF and cognitive function.^1^ Both lowest and highest quintiles of LVEF were associated with impaired cognitive function. In contrast, higher cardiac index was shown to be associated with higher cognitive function.^6^ Higher LV mass was also shown to predict cognitive impairment and dementia in the MESA (Multi-Ethnic Study of Atherosclerosis).^28^ Likewise, higher global longitudinal strain was associated with lower cognitive performance in Vanderbilt Memory and Aging Project.^2^ However, these studies were limited to older cohorts with a mean age above 60 years. Other non-U.S. cohorts of older adults from Iceland and the United Kingdom have shown conflicting evidence regarding the relationship between LVEF and brain function, but the proportion of ethnic minorities was very small among those studies.^7^^,^^8^

Few studies have investigated the relationship between cardiac structure and cognitive function. Analysis from the Coronary Artery Risk Development in Young Adults reported that higher LV mass index associated with lower cognitive function during midlife.^29^ Our study extended the findings and demonstrated stronger associations between an increase in LV mass, concentricity, or LV E_cc_ and an increase in WMH in Black vs non-Black participants. This is an important finding as it may explain mechanisms underlying an accelerated brain aging beginning from midlife among Black adults when compared to other racial/ethnic groups as evidenced by increased WMH shown in a recent study.^21^ In addition, an increasing number of studies have showed a paradoxically lower amyloid brain deposit among Black individuals as evidenced by positron emission tomography scan despite having higher prevalence of ApoE-ε4 alleles when compared to White individuals.^9^^,^^10^ Thus, other factors are likely responsible for the racial/ethnic disparities in the prevalence of dementia and Alzheimer disease in this population. Our finding suggests a potential role for optimal cardiovascular health to preserve brain function in this racial/ethnic subgroup which is disproportionately affected by Alzheimer disease.^30^

On the other hand, ApoE-ε4 allele carrier status did not modify the relationship between LV function/structure and MoCA or brain structure. Our findings are in contrast with the results from the Vanderbilt Memory and Aging project which showed that lower cardiac output was associated with poorer cognitive performances in ApoE-ε4 allele carriers but not among noncarriers.^31^ Although factors underlying these divergent results are unknown, the mean age of our cohort is on average more than 20 years younger and more ethnically diverse than previous studies. Moreover, statistical power for subgroup analyses was limited. Thus, additional studies are needed to determine whether ApoE status influences associations between the cardiac and brain axes and whether this differs by race/ethnicity.

The strengths of our study include the use of a highly accurate technique of cMRI in determining cardiac structure and function as well as myocardial strain, which is shown to be a more sensitive marker for LV contractility^32^ and predictor of cardiovascular prognosis than LVEF alone,^33^^,^^34^ in a multiethnic population without overt cardiovascular disease. Our study is limited by the use of MoCA alone as the tool for assessment of cognitive function. The cross-sectional design also limits the ability to determine causal effects. Racial/ethnic data were collected by self-reported only which may be the result of multiple factors including social determinants of health or genetic variation and should not be used to imply underlying genetic effects alone.^35^ Nevertheless, our data suggest a link between subclinical abnormalities in cardiac structure and function with a higher burden of WMH after adjusting for physical activity, particularly among Black participants, the population at the highest risk for ADRD. Future studies are needed to determine if strategies to maintain normal cardiac structure and function during midlife offers protection against cognitive decline in late life.

## Conclusions

Subclinical cardiac dysfunction was associated with lower cognitive function. Moreover, increased LV mass, concentric remodeling, and higher LV E_cc_ were associated with higher burden of WMH, particularly among Black individuals.

## Funding support and author disclosures

The Dallas Heart Study was funded by the Donald W. Reynolds Foundation and was partially supported by the National Center for Advancing Translational Sciences of the National Institutes of Health under award Number UL1TR001105. Dr Vongpatanasin is supported by the 10.13039/100007914UT Southwestern O’Brien Kidney Center, the Charles and Jane Pak Center for Mineral Metabolism and Clinical Research: R01 AG057571. All other authors have reported that they have no relationships relevant to the contents of this paper to disclose.

PERSPECTIVES **COMPETENCY IN MEDICAL KNOWLEDGE:** Subclinical cardiac dysfunction was associated with lower cognitive function. Additionally, subclinical cardiac remodeling was associated with subclinical brain injury. These findings highlight the importance of maintaining optimal cardiovascular health to aid in maintaining brain health.

**TRANSLATIONAL OUTLOOK:** Further studies are needed to determine if strategies to maintain normal cardiac structure and function during midlife offers protection against cognitive decline in late life.
