# Supplementary material for: Associations Between Cardiac Function and Brain Health in Diverse Middle-Aged Adults: The Dallas Heart Study-2
Source: JACC Adv. 2023 Dec 22;3(2):100777. doi: 10.1016/j.jacadv.2023.100777 (PMC11198548; doi:10.1016/j.jacadv.2023.100777)
Supplement: Supplemental Tables 1-8 [file mmc1.docx]

**Supplemental Table 1.** Associations between cardiac structure/function and total Montreal Cognitive Assessment score

| **Cardiac Variable** | **Model 1** | | **Model 2** | | **Model 3** | |
| --- | --- | --- | --- | --- | --- | --- |
|  | **β (95% CI)^a^** | **p-value** | **β (95% CI)^a^** | **p-value** | **β (95% CI)^a^** | **p-value** |
| LVEF | 0.01 (-0.04, 0.06) | 0.652 | 0.05 (-0.01, 0.10) | 0.110 | 0.06 (0.002, 0.12) | 0.044 ^b^ |
| LAV | -0.04 (-0.12, 0.05) | 0.400 | 0.13 (0.008, 0.25) | 0.038^b^ | 0.11 (-0.02, 0.23) | 0.109 |
| Cardiac output | -0.009 (-0.06, 0.04) | 0.737 | 0.01 (-0.05, 0.08) | 0.704 | 0.01 (-0.06, 0.08) | 0.753 |
| LV concentricity | -0.02 (-0.07, 0.03) | 0.499 | -0.03 (-0.10, 0.03) | 0.307 | -0.02 (-0.08, 0.05) | 0.600 |
| Stroke Volume | 0.01 (-0.04, 0.07) | 0.705 | 0.07 (0.004, 0.14) | 0.038 ^b^ | 0.07 (-0.006, 0.14) | 0.071 |
| EDV | -0.003 (-0.06, 0.05) | 0.925 | 0.02 (-0.05, 0.09) | 0.513 | 0.007 (-0.07, 0.08) | 0.857 |
| ESV | -0.01 (-0.07, 0.04) | 0.587 | -0.03 (-0.10, 0.03) | 0.314 | -0.05 (-0.12, 0.02) | 0.137 |
| HR | -0.03 (-0.07, 0.02) | 0.234 | -0.05 (-0.11, 0.005) | 0.077 | -0.05 (-0.11, 0.01) | 0.103 |
| LV mass | -0.008 (-0.07, 0.06) | 0.802 | -0.007 (-0.09, 0.08) | 0.876 | -0.003 (-0.09, 0.09) | 0.949 |
| LVH (by BSA) | -0.007 (-0.05, 0.04) | 0.769 | -0.01 (-0.07, 0.04) | 0.622 | -0.03 (-0.09, 0.03) | 0.366 |
| Peak systolic strain | -0.04 (-0.09, 0.01) | 0.096 | -0.06 (-0.11, 0.003) | 0.065 | -0.05 (-0.12, 0.01) | 0.102 |

^a^ β and 95% confidence interval values are presented as standardized β.

^b^ indicates statistical significance.

**Model 1:** Age, sex, race, BSA. **Model 2**: Model 1 + SBP, antihypertensive, atrial fibrillation, diabetes, smoking, eGFR, and education levels. **Model 3**: Model 2 + MVPA

**Abbreviations:** BSA: body surface area; EDV: end-diastolic volume; ESV: end-systolic volume; HR: heart rate; LAV: left atrial volume; LV: left ventricle; LVEF: left ventricular ejection fraction; LVH: left ventricular hypertrophy, MVPA: moderate-to-vigorous physical activity.

**Supplemental Table 2.** Associations between white matter hyperintensity volume normalized to total cranial volume and cardiac structure/function

| **Cardiac Variable** | **Model 1** | | **Model 2** | | **Model 3** | |
| --- | --- | --- | --- | --- | --- | --- |
|  | **β (95% CI)^a^** | **p-value** | **β (95% CI)^a^** | **p-value** | **β (95% CI)^a^** | **p-value** |
| LVEF | -0.03 (-0.07, 0.01) | 0.163 | -0.06 (-0.12, 0.001) | 0.054 | -0.07 (-0.14, -0.009) | 0.027 ^b^ |
| LAV | 0.01 (-0.06, 0.09) | 0.753 | -0.08 (-0.21, 0.05) | 0.209 | -0.05 (-0.19, 0.08) | 0.443 |
| Cardiac output | 0.06 (0.02, 0.11) | 0.009 ^b^ | 0.02 (-0.06, 0.09) | 0.682 | -0.006 (-0.08, 0.07) | 0.883 |
| LV concentricity | 0.10 (0.06, 0.14) | <0.0001 ^b^ | 0.08 (0.02, 0.15) | 0.013 ^b^ | 0.10 (0.03, 0.17) | 0.004 ^b^ |
| Stroke Volume | 0.03 (-0.02, 0.08) | 0.229 | -0.04 (-0.11, 0.04) | 0.327 | -0.04 (-0.12, 0.04) | 0.347 |
| EDV | 0.05 (-0.001, 0.10) | 0.057 | 0.003 (-0.07, 0.08) | 0.929 | 0.01 (-0.07, 0.09) | 0.740 |
| ESV | 0.05 (-0.00007, 0.09) | 0.051 | 0.04 (-0.03, 0.11) | 0.229 | 0.06 (-0.01, 0.13) | 0.119 |
| HR | 0.04 (-0.003, 0.08) | 0.068 | 0.05 (-0.006, 0.12) | 0.080 | 0.04 (-0.03, 0.11) | 0.251 |
| LV mass | 0.17 (0.11, 0.23) | <0.0001 ^b^ | 0.09 (0.001, 0.18) | 0.048 ^b^ | 0.13 (0.03, 0.23) | 0.009 ^b^ |
| LVH (by BSA) | 0.11 (0.07, 0.15) | <0.0001 ^b^ | 0.11 (0.05, 0.17) | 0.0002 ^b^ | 0.11 (0.04, 0.17) | 0.002 ^b^ |
| Peak systolic strain | 0.07 (0.03, 0.11) | 0.0005 ^b^ | 0.06 (0.003, 0.13) | 0.040 ^b^ | 0.08 (0.01, 0.15) | 0.020 ^b^ |

^a^ β and 95% confidence interval values are presented as standardized β.

^b^ indicates statistical significance.

**Model 1:** Age, sex, race, BSA. **Model 2**: Model 1 + SBP, antihypertensive, atrial fibrillation, diabetes mellitus, smoking, eGFR, and education levels. **Model 3**: Model 2 + time spent in MVPA

**Abbreviations:** BSA: body surface area; EDV: end-diastolic volume; ESV: end-systolic volume; HR: heart rate; LAV: left atrial volume; LV: left ventricle; LVEF: left ventricular ejection fraction; LVH: left ventricular hypertrophy; MVPA = moderate-to-vigorous physical activity.

**Supplemental Table 3**. Association between cardiac structure/function and Montreal Cognitive Assessment stratified by race

| **Cardiac Variable** | **Black** | | **Non-Black** | | **Interaction**  **p-value** |
| --- | --- | --- | --- | --- | --- |
|  | **β (95% CI)^a^** | **p-value** | **β (95% CI)^a^** | **p-value** |  |
| LVEF | 0.08 (-0.01, 0.18) | 0.085 | 0.03 (-0.06, 0.12) | 0.489 | 0.580 |
| LAV | 0.19 (0.01, 0.37) | 0.039 ^b^ | -0.08 (-0.3, 0.14) | 0.479 | 0.349 |
| Cardiac output | 0.02 (-0.09, 0.13) | 0.731 | -0.01 (-0.12, 0.10) | 0.850 | 0.537 |
| LV concentricity | -0.06 (-0.16, 0.04) | 0.257 | 0.03 (-0.07, 0.13) | 0.566 | 0.605 |
| Stroke Volume | 0.08 (-0.04, 0.19) | 0.180 | 0.05 (-0.06, 0.16) | 0.412 | 0.673 |
| EDV | -0.02 (-0.13, 0.09) | 0.751 | 0.04 (-0.08, 0.16) | 0.500 | 0.683 |
| ESV | -0.09 (-0.19, 0.01) | 0.088 | 0.01 (-0.09, 0.12) | 0.800 | 0.399 |
| HR | -0.05 (-0.14, 0.05) | 0.320 | -0.06 (-0.15, 0.04) | 0.235 | 0.740 |
| LV mass | -0.07 (-0.20, 0.06) | 0.283 | 0.08 (-0.06, 0.22) | 0.272 | 0.678 |
| LVH (by BSA) | -0.07 (-0.16, 0.03) | 0.166 | 0.03 (-0.06, 0.12) | 0.515 | 0.429 |
| Peak systolic strain | -0.15 (-0.24, -0.05) | 0.004 ^b^ | 0.05 (-0.04, 0.14) | 0.270 | 0.009 ^b^ |

^a^ β and 95% confidence interval values are presented as standardized β.

^b^ indicates statistical significance.

**Covariates**: Age, sex, BSA, SBP, antihypertensive, atrial fibrillation, diabetes, smoking, eGFR, and education levels, and MVPA

**Abbreviations:** BSA: body surface area; EDV: end-diastolic volume; ESV: end-systolic volume; HR: heart rate; LAV: left atrial volume; LV: left ventricle; LVEF: left ventricular ejection fraction; LVH: left ventricular hypertrophy; MVPA = moderate-to-vigorous physical activity.

**Supplemental Table 4**. Association between cardiac structure/function and white matter hyperintensity volume normalized to total cranial volume stratified by race/ethnicity

| **Cardiac Variable** | **Black** | | **Non-Black** | | **Interaction**  **p-value** |
| --- | --- | --- | --- | --- | --- |
|  | **β (95% CI)^a^** | **p-value** | **β (95% CI)^a^** | **p-value** |  |
| LVEF | -0.11 (-0.21, -0.02) | 0.018 ^b^ | -0.01 (-0.11, 0.08) | 0.806 | 0.160 |
| LAV | -0.12 (-0.31, 0.07) | 0.233 | 0.1 (-0.12, 0.31) | 0.381 | 0.819 |
| Cardiac output | -0.11 (-0.22, -0.004) | 0.043 ^b^ | 0.11 (0.001, 0.22) | 0.048 ^b^ | 0.055 |
| LV concentricity | 0.23 (0.13, 0.32) | <0.0001 ^b^ | -0.11 (-0.21, -0.009) | 0.033 ^b^ | <0.0001 ^b^ |
| Stroke Volume | -0.17 (-0.28, -0.06) | 0.003 ^b^ | 0.13 (0.02, 0.25) | 0.026 ^b^ | 0.025 ^b^ |
| EDV | -0.08 (-0.19, 0.03) | 0.175 | 0.13 (0.02, 0.25) | 0.028 ^b^ | 0.172 |
| ESV | 0.04 (-0.06, 0.14) | 0.467 | 0.08 (-0.03, 0.18) | 0.163 | 0.854 |
| HR | 0.04 (-0.05, 0.13) | 0.410 | 0.02 (-0.08, 0.11) | 0.729 | 0.944 |
| LV mass | 0.17 (0.05, 0.30) | 0.008 ^b^ | -0.01 (-0.16, 0.14) | 0.880 | 0.030 ^b^ |
| LVH (by BSA) | 0.14 (0.05, 0.23) | 0.004 ^b^ | -0.00003 (-0.09, 0.09) | 0.999 | 0.326 |
| Peak systolic strain | 0.11 (0.02, 0.21) | 0.023 ^b^ | 0.02 (-0.07, 0.11) | 0.679 | 0.156 |

^a^ β and 95% confidence interval values are presented as standardized β.

^b^ indicates statistical significance.

**Covariates**: Age, sex, BSA, SBP, antihypertensive, atrial fibrillation, diabetes, smoking, eGFR, and education levels, and MVPA

**Abbreviations:** BSA: body surface area; EDV: end-diastolic volume; ESV: end-systolic volume; HR: heart rate; LAV: left atrial volume; LV: left ventricle; LVEF: left ventricular ejection fraction; LVH: left ventricular hypertrophy; MVPA = moderate-to-vigorous physical activity.

**Supplemental Table 5.** Association between cardiac structure/function and Montreal Cognitive Assessment score stratified by ApoE-ε4 genotype (adjusted for model 3 covariates)

| **Cardiac Variable** | **ApoE negative (n=764)** | | **ApoE positive (n=362)** | | **Interaction**  **p-value** |
| --- | --- | --- | --- | --- | --- |
|  | **β (95% CI)^a^** | **p-value** | **β (95% CI)^a^** | **p-value** |  |
| LVEF | 0.06 (-0.03, 0.16) | 0.196 | 0.06 (-0.06, 0.18) | 0.343 | 0.804 |
| LAV | 0.13 (-0.03, 0.29) | 0.102 | 0.006 (-0.24, 0.25) | 0.962 | 0.055 |
| Cardiac output | 0.01 (-0.10, 0.12) | 0.846 | -0.18 (-0.31, -0.04) | 0.011 ^b^ | 0.031 ^b^ |
| LV concentricity | 0.004 (-0.10, 0.11) | 0.939 | 0.02 (-0.12, 0.16) | 0.773 | 0.879 |
| Stroke Volume | 0.05 (-0.06, 0.17) | 0.390 | -0.08 (-0.23, 0.07) | 0.293 | 0.121 |
| EDV | -0.004 (-0.12, 0.11) | 0.950 | -0.11 (-0.25, 0.04) | 0.142 | 0.153 |
| ESV | -0.05 (-0.16, 0.05) | 0.326 | -0.1 (-0.23, 0.03) | 0.151 | 0.413 |
| HR | -0.02 (-0.12, 0.08) | 0.675 | -0.15 (-0.26, -0.03) | 0.017 ^b^ | 0.146 |
| LV mass | -0.006 (-0.15, 0.14) | 0.929 | -0.15 (-0.33, 0.03) | 0.101 | 0.186 |
| LVH (by BSA) | -0.03 (-0.12, 0.07) | 0.608 | -0.05 (-0.19, 0.09) | 0.470 | 0.488 |
| Peak systolic strain | 0.009 (-0.09, 0.11) | 0.858 | -0.16 (-0.28, -0.03) | 0.016 ^b^ | 0.221 |

^a^ β and 95% confidence interval values are presented as standardized β.

^b^ indicates statistical significance.

**Covariates:** Age, sex, race, BSA, SBP, antihypertensive, diabetes, smoking, eGFR, and education levels, and MVPA

**Abbreviations:** BSA: body surface area; EDV: end-diastolic volume; ESV: end-systolic volume; HR: heart rate; LAV: left atrial volume; LV: left ventricle; LVEF: left ventricular ejection fraction; LVH: left ventricular hypertrophy; MVPA = moderate-to-vigorous physical activity.

**Supplemental Table 6.** Association between cardiac structure/function and white matter hyperintensity volume normalized to total cranial volume, stratified by ApoE-ε4 genotype (adjusted for model 3 covariates)

| **Cardiac Variable** | **ApoE negative** | | **ApoE positive** | | **Interaction**  **p-value** |
| --- | --- | --- | --- | --- | --- |
|  | **β (95% CI)^a^** | **p-value** | **β (95% CI)^a^** | **p-value** |  |
| LVEF | -0.13 (-0.23, -0.02) | 0.015 ^b^ | -0.07 (-0.23, 0.08) | 0.351 | 0.821 |
| LAV | -0.03 (-0.20, 0.14) | 0.719 | -0.13 (-0.41, 0.14) | 0.342 | 0.758 |
| Cardiac output | 0.09 (-0.02, 0.21) | 0.115 | -0.06 (-0.23, 0.11) | 0.504 | 0.115 |
| LV concentricity | 0.16 (0.05, 0.27) | 0.004 ^b^ | 0.01 (-0.16, 0.18) | 0.910 | 0.117 |
| Stroke Volume | 0.007 (-0.11, 0.13) | 0.910 | 0.06 (-0.12, 0.25) | 0.509 | 0.653 |
| EDV | 0.09 (-0.03, 0.21) | 0.138 | 0.09 (-0.09, 0.27) | 0.327 | 0.886 |
| ESV | 0.13 (0.02, 0.24) | 0.019 ^b^ | 0.09 (-0.08, 0.25) | 0.301 | 0.897 |
| HR | 0.10 (0.003, 0.21) | 0.045 ^b^ | -0.11 (-0.26, 0.04) | 0.160 | 0.006 ^b^ |
| LV mass | 0.29 (0.14, 0.44) | 0.0002 ^b^ | 0.19 (-0.04, 0.42) | 0.111 | 0.685 |
| LVH (by BSA) | 0.12 (0.02, 0.22) | 0.016 ^b^ | 0.21 (0.03, 0.38) | 0.022 ^b^ | 0.852 |
| Peak systolic strain | 0.17 (0.07, 0.28) | 0.001 ^b^ | 0.07 (-0.09, 0.22) | 0.399 | 0.250 |

^a^ β and 95% confidence interval values are presented as standardized β.

^b^ indicates statistical significance.

**Covariates**: Age, sex, race, BSA, SBP, antihypertensive, diabetes, smoking, eGFR, and education levels, and MVPA

**Abbreviations:** BSA: body surface area; EDV: end-diastolic volume; ESV: end-systolic volume; HR: heart rate; LAV: left atrial volume; LV: left ventricle; LVEF: left ventricular ejection fraction; LVH: left ventricular hypertrophy; MVPA = moderate-to-vigorous physical activity.

**Supplemental Table 7.** Association between cardiac structure/function and Montreal Cognitive Assessment score stratified by ApoE-ε4 genotype

| **Cardiac Variable** | **ApoE negative** | | **ApoE positive** | | **Interaction**  **p-value** |
| --- | --- | --- | --- | --- | --- |
|  | **β (95% CI)^a^** | **p-value** | **β (95% CI)^a^** | **p-value** |  |
| LVEF | 0.06 (-0.03, 0.16) | 0.200 | 0.06 (-0.06, 0.18) | 0.334 | 0.816 |
| LAV | 0.13 (-0.03, 0.29) | 0.112 | 0.007 (-0.24, 0.25) | 0.958 | 0.052 |
| Cardiac output | 0.007 (-0.10, 0.12) | 0.901 | -0.18 (-0.31, -0.04) | 0.011 ^b^ | 0.034 ^b^ |
| LV concentricity | 0.004 (-0.10, 0.11) | 0.939 | 0.02 (-0.12, 0.16) | 0.765 | 0.894 |
| Stroke Volume | 0.05 (-0.07, 0.16) | 0.419 | -0.08 (-0.24, 0.07) | 0.279 | 0.127 |
| EDV | -0.006 (-0.12, 0.11) | 0.916 | -0.11 (-0.25, 0.03) | 0.133 | 0.159 |
| ESV | -0.05 (-0.16, 0.05) | 0.317 | -0.10 (-0.23, 0.03) | 0.145 | 0.421 |
| HR | -0.02 (-0.12, 0.07) | 0.645 | -0.15 (-0.27, -0.03) | 0.018 ^b^ | 0.150 |
| LV mass | -0.009 (-0.15, 0.14) | 0.905 | -0.16 (-0.34, 0.03) | 0.096 | 0.188 |
| LVH (by BSA) | -0.03 (-0.12, 0.07) | 0.574 | -0.05 (-0.19, 0.09) | 0.467 | 0.490 |
| Peak systolic strain | 0.009 (-0.09, 0.11) | 0.860 | -0.16 (-0.28, -0.03) | 0.017 ^b^ | 0.216 |

^a^ β and 95% confidence interval values are presented as standardized β.

^b^ indicates statistical significance.

**Covariates:** Age, sex, race, BSA, SBP, antihypertensive, atrial fibrillation, diabetes, smoking, eGFR, and education levels, and MVPA

**Abbreviations:** BSA: body surface area; EDV: end-diastolic volume; ESV: end-systolic volume; HR: heart rate; LAV: left atrial volume; LV: left ventricle; LVEF: left ventricular ejection fraction; LVH: left ventricular hypertrophy; MVPA = moderate-to-vigorous physical activity.

**Supplemental Table 8.** Association between cardiac structure/function and white matter hyperintensity volume normalized to total cranial volume, stratified by ApoE-ε4 genotype

| **Cardiac Variable** | **ApoE negative** | | **ApoE positive** | | **Interaction**  **p-value** |
| --- | --- | --- | --- | --- | --- |
|  | **β (95% CI)^a^** | **p-value** | **β (95% CI)^a^** | **p-value** |  |
| LVEF | -0.13 (-0.23, -0.03) | 0.015 ^b^ | -0.07 (-0.23, 0.08) | 0.367 | 0.755 |
| LAV | -0.03 (-0.20, 0.14) | 0.768 | -0.13 (-0.40, 0.15) | 0.367 | 0.803 |
| Cardiac output | 0.10 (-0.01, 0.22) | 0.075 | -0.06 (-0.23, 0.11) | 0.489 | 0.091 |
| LV concentricity | 0.16 (0.05, 0.26) | 0.004 ^b^ | 0.01 (-0.16, 0.18) | 0.897 | 0.133 |
| Stroke Volume | 0.02 (-0.10, 0.13) | 0.804 | 0.06 (-0.13, 0.24) | 0.538 | 0.719 |
| EDV | 0.10 (-0.02, 0.22) | 0.106 | 0.09 (-0.09, 0.27) | 0.350 | 0.970 |
| ESV | 0.14 (0.03, 0.25) | 0.015 ^b^ | 0.08 (-0.08, 0.25) | 0.320 | 0.818 |
| HR | 0.11 (0.009, 0.21) | 0.032 ^b^ | -0.11 (-0.26, 0.04) | 0.168 | 0.005 ^b^ |
| LV mass | 0.30 (0.15, 0.44) | 0.0001 ^b^ | 0.18 (-0.05, 0.42) | 0.119 | 0.655 |
| LVH (by BSA) | 0.13 (0.03, 0.23) | 0.011 ^b^ | 0.21 (0.03, 0.38) | 0.023 ^b^ | 0.853 |
| Peak systolic strain | 0.17 (0.07, 0.27) | 0.001 ^b^ | 0.07 (-0.09, 0.23) | 0.385 | 0.271 |

^a^ β and 95% confidence interval values are presented as standardized β .

^b^ indicates statistical significance.

**Covariates**: Age, sex, race, BSA, SBP, antihypertensive, atrial fibrillation, diabetes, smoking, eGFR, and education levels, and MVPA

**Abbreviations:** BSA: body surface area; EDV: end-diastolic volume; ESV: end-systolic volume; HR: heart rate; LAV: left atrial volume; LV: left ventricle; LVEF: left ventricular ejection fraction; LVH: left ventricular hypertrophy; MVPA = moderate-to-vigorous physical activity.
